# *Bacillus subtilis* Two-Component System Sensory Kinase DegS Is Regulated by Serine Phosphorylation in Its Input Domain

**DOI:** 10.1371/journal.pone.0014653

**Published:** 2011-02-03

**Authors:** Carsten Jers, Ahasanul Kobir, Elsebeth Oline Søndergaard, Peter Ruhdal Jensen, Ivan Mijakovic

**Affiliations:** 1 Center for Systems Microbiology, Technical University of Denmark, Lyngby, Denmark; 2 Micalis, AgroParisTech/Institut National de la Recherche Agronomique, Jouy en Josas, France; Texas A&M University, United States of America

## Abstract

*Bacillus subtilis* two-component system DegS/U is well known for the complexity of its regulation. The cytosolic sensory kinase DegS does not receive a single predominant input signal like most two-component kinases, instead it integrates a wide array of metabolic inputs that modulate its activity. The phosphorylation state of the response regulator DegU also does not confer a straightforward “on/off” response; it is fine-tuned and at different levels triggers different sub-regulons. Here we describe serine phosphorylation of the DegS sensing domain, which stimulates its kinase activity. We demonstrate that DegS phosphorylation can be carried out by at least two *B. subtilis* Hanks-type kinases *in vitro*, and this stimulates the phosphate transfer towards DegU. The consequences of this process were studied *in vivo*, using phosphomimetic (Ser76Asp) and non-phosphorylatable (Ser76Ala) mutants of DegS. In a number of physiological assays focused on different processes regulated by DegU, DegS S76D phosphomimetic mutant behaved like a strain with intermediate levels of DegU phosphorylation, whereas DegS S76A behaved like a strain with lower levels of DegU phophorylation. These findings suggest a link between DegS phosphorylation at serine 76 and the level of DegU phosphorylation, establishing this post-translational modification as an additional trigger for this two-component system.

## Introduction

Two-component systems are a ubiquitous means of signal transduction in bacteria [1]. The first, signal-receiving component is a sensory histidine kinase that is triggered by a stimulus binding or otherwise affecting its sensing domain. Upon activation, the histidine kinase autophosphorylates on a histidine residue, and thereafter transfers the phosphate to a specific aspartate residue of its cognate response regulator. Phosphorylation of the response regulator, in turn, triggers its regulatory role, which is in most cases transcriptional regulation via binding of a specific DNA sequence. The histidine kinases of the two-component systems are known to be highly specific, i.e. exhibiting low level of cross-talk with non-cognate response regulators [2]. Another major group of bacterial kinases involved in signal transduction is the Hanks type serine/threonine kinases [3], [4]. Hanks type kinases and two-component histidine kinases are sometimes found fusioned in a single polypeptide in *Cyanobacteria* [5], however, very few cases of crosstalk between these two protein families have been reported so far. Two recent studies pointed out that serine/threonine kinases can phosphorylate two-component response regulators: StkP from *Streptococcus pneumoniae* phosphorylates the orphan response regulator RitR [6] and serine-threonine kinase Stk1 phosphorylates and thereby abolishes the activity of the response regulator CovR in Group B *Streptococcus* [7]. Interestingly, a recent phosphoproteomic study in *Bacillus subtilis*, identified the two-component system histidine kinase DegS as being phosphorylated on the residue serine76, located in the signal sensing domain [8]. This implied the existence of a new type of crosstalk between two phosphorylation systems, namely one in which a presently unknown serine kinase would phosphorylate the two-component sensory histidine kinase.

Early mutational studies of the DegS/U two component system established that the response regulator binds DNA sequences and regulates expression of specific genes both in its phosphorylated and unphosphorylated state. This was exemplified by reciprocal effects of the two forms of DegU on exoprotease production and competence [9]. The importance of DegS/U was further underscored in two microarray experiments that independently demonstrated a total of 135 transcriptional units regulated either directly or indirectly by this two-component system [10], [11]. Only 22 transcriptional units were identified in the overlap between the two studies, which could indicate an even larger regulon. Whereas the initial studies of DegS/U in the laboratory strain 168 mainly focused on competence and exoprotease production, more recent studies using an undomesticated *B. subtilis* strain demonstrated that DegS/U also affects motility, complex colony and biofilm formation. The regulation was shown to depend on the discrete levels of DegU phosphorylation, in a manner far more subtle than a simple on/off switch [12], [13]. It is now well established that DegS/U system plays an important role in the transition growth phase where it receives many inputs which regulate *degSU* transcription or modulate the activity of synthesized DegS/U proteins. The *degSU* genes are transcribed as an operon and *degU* is itself transcribed from two additional promoters: one activated by DegU∼P and the other by nitrogen starvation [9], [13]–[16]. The signal-sensing domain of DegS interacts with the SMC-ScpA-ScpB protein complex, involved in chromosome segregation, which inhibits the kinase activity of DegS [17]. Similarly, the DNA-binding activity of DegU is inhibited by RapG and this inhibition is counteracted by PhrG in response to increased cell density [18]. DegS/U activity is further modulated by two small regulatory peptides, DegQ and DegR. DegQ enhances the phosphotransfer from DegS∼P to DegU but does not protect DegU∼P from dephosphorylation [13]. The latter is accomplished by DegR that stabilises DegU∼P [19]. DegQ is synthesized in response to quorum sensing via ComPA and has been hypothesised to be a determinant for the transition from motile to sessile-growth state [13]. *degR* expression is SigD dependent and peaks in late exponential phase, but the physiological role remains elusive [20].

Despite the fact that the DegS/U two-component system is submitted to an elaborate control at both transcriptional and protein level, no specific DegS-activating signal has so far been proposed. Most two-component system histidine kinases are transmembrane proteins, presumably activated by extracellular signals via their N-terminal signal-sensing domains protruding from the cell surface [21]. By contrast, DegS is a cytosolic protein and hence responds to, and integrates several cytosolic signals, some of which have been listed above. In this study, we examined the possibility that phosphorylation of DegS on serine 76 residue could represent a novel input for this regulatory system. We demonstrated that the specific phosphorylation of this residue by the Hanks kinase YbdM stimulates phosphotransfer to DegU *in vitro*, and a phosphomimetic mutant of DegS leads to an increased DegU∼P pool, and influences the transcription of key DegU-dependent promoters *in vivo*.

## Results and Discussion

### DegS is phosphorylated *in vitro* by *B. subtilis* Hanks-type serine/threonine kinases

**Figure 1:**
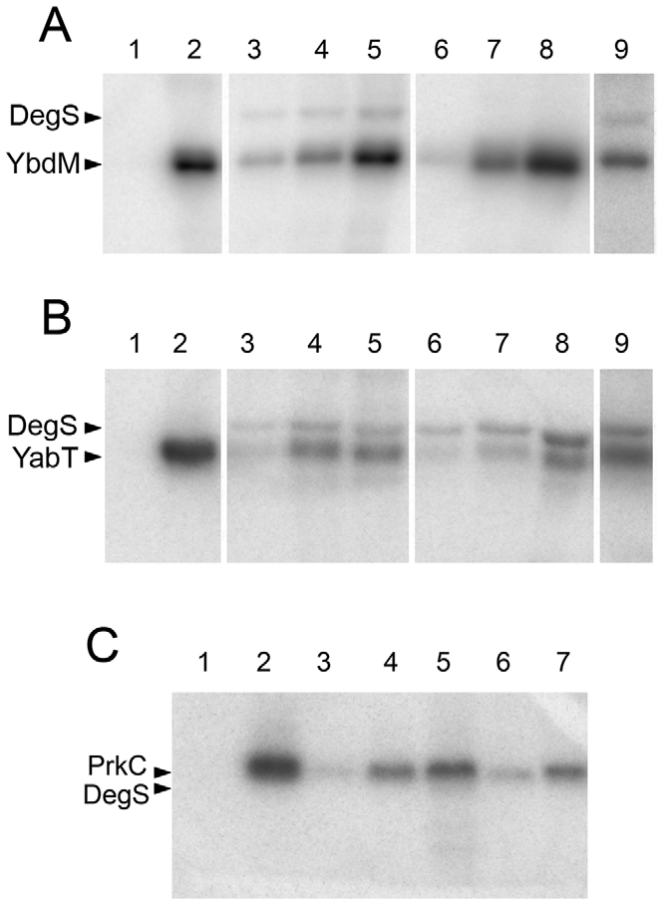
Phosphorylation of DegS by Hanks type kinases *in vitro*. Autoradiography of SDS-Polyacrylamide gels with proteins that had been incubated with ^32^P-γ-ATP. Gels were treated by boiling in acid to remove phospho-histidine signals (A) Phosphorylation of DegS by the kinase YbdM. Lanes 1 and 2 are controls, with DegS alone and YbdM alone, respectively. Lanes 3–5 show phosphorylation of DegS by YbdM for 15, 30 and 60 min, respectively. Lanes 6–8 show phosphorylation of DegS S76A by YbdM for 15, 30 and 60 min, respectively. Lane 9 shows the equivalent of the reaction from lane 5, after desalting to remove the ATP and a 2 h incubation to test for phosphatase activity. (B) Phosphorylation of DegS by the kinase YabT. Lanes 1 and 2 are controls, with DegS alone and YabT alone, respectively. Lanes 3–5 show phosphorylation of DegS by YabT for 15, 30 and 60 min, respectively. Lanes 6–8 show phosphorylation of DegS S76A by YabT for 15, 30 and 60 min, respectively. Lane 9 shows the equivalent of the reaction from lane 5, after desalting to remove the ATP and a 2 h incubation to test for phosphatase activity. (C) Phosphorylation of DegS by the kinase PrkC. Lanes 1 and 2 are controls, with DegS alone and PrkC alone, respectively. Lanes 3–5 contain the reactions where PrkC concentration has been varied (2, 4 and 10 nM, respectively), and in lanes 6–7 the pH has been varied (pH 7 and pH 8, respectively).

**Figure 2:**
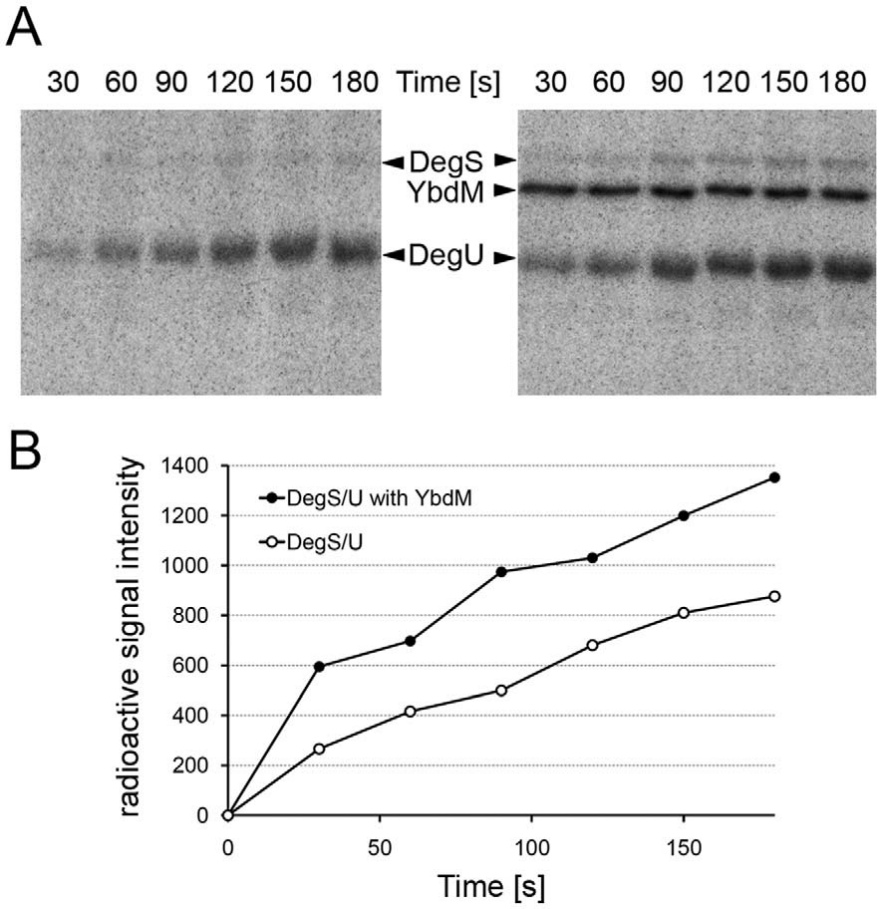
Phosphorylation of DegS by YbdM stimulates phosphotransfer to DegU *in vitro*. (A) Autoradiography of SDS-Polyacrylamide gels with proteins that had been incubated with ^32^P-γ-ATP. Gels were not treated by boiling in acid, so the phospho-histidine and phospho-aspartate signals are preserved. Efficiency of phosphotransfer of wild-type DegS to DegU (gel on the left) is compared to that of DegS that had been preincubated with YbdM, 50 µM non-labelled ATP and 5 mM MgCl_2_ for 3 h (gel on the right). (B) Quantification of DegU phosphorylation signals on both gels.

In order to characterize the serine 76 phosphorylation of DegS, we first asked the question whether this phosphorylation is auto-catalyzed or it requires another kinase. To answer this question we carried out *in vitro* phosphorylation experiments with purified DegS and ^32^P-γ-ATP. Phospho-histidine and phospho-serine can be easily distinguished, the latter being stable in acidic conditions and resistant to heat. Since the entire radioactive label present on autophosphorylated DegS was removed by acid and heat treatment (figure 1), we concluded that DegS was incapable of autophosphorylating on serine. There are a number of poorly characterized serine/threonine kinases in *B. subtilis*, mainly belonging to the family of Hanks-type kinases [22]. The most extensively characterized of those is the kinase PrkC that has recently been shown to participate in signalling underlying spore germination [23]. We purified the three Hanks-type kinases PrkC, YabT and YbdM, and a putative kinase PrkA to test their ability to phosphorylate DegS *in vitro*. PrkA (data not shown) and PrkC were unable to phosphorylate DegS, whereas both YbdM and YabT tested positive for DegS phosphorylation (figure 1). In order to verify whether serine 76 is indeed the residue phosphorylated by these kinases, we constructed a mutant protein with a non-phosphorylatable replacement DegS S76A. Phosphorylation of DegS S76A by YbdM was completely abolished, suggesting that it is the major phosphorylation site, and that the kinase YbdM is specific for this site (figure 1A). In the same assay, phosphorylation of DegS S76A by YabT was as efficient as that of the wild type (figure 1B). This situation is not unprecedented [24], and residual phosphorylation in such case could be due to a presently unknown secondary site, or the lack of specificity exhibited by the kinase under *in vitro* conditions. Our conclusion was that *in vitro* at least two different Hanks-type kinases can phosphorylate DegS, of which YbdM is specific for the residue serine 76. DegS is also known to exhibit phosphatase activity, so we tested whether serine phosphorylation of DegS could be removed by the protein itself. For this, we removed the ATP from the phosphorylation reactions catalyzed by YbdM and YabT, and allowed the dephosphorylation reaction to occur for 2 h. The radiolabel on DegS remained stable, indicating the absence of phospho-serine phosphatase activity (figure 1A and 1B, lanes 9). The *B. subtilis* serine/threonine kinase-encoding genes *yabT*, *ybdM* and *prkA* have been reported to belong to the sigma F, G and E regulon respectively [25], [26] hence linking them to sporulation specific processes. However, a recent transcriptomics study [27] identified *yabT*, *ybdM* and *prkC* as transcribed in the exponential growth phase, which makes it highly probable that these kinases are present in the transition phase when DegS activity is triggered. Since YbdM appeared to be the the most specific kinase for DegS serine 76, we tested the effect of YbdM-dependent DegS phosphorylation on the efficiency of phosphotransfer from DegS to DegU. To this end, we first incubated DegS with non-labelled ATP, MgCl_2_ and YbdM for 3 hrs, and we also prepared a control sample of DegS incubated for exactly the same time with ATP and MgCl_2_, but without YbdM. The DegS sample pre-incubated with YbdM was more efficient in phosphorylating DegU, especially in the first 60 s period which corresponds to the theoretical reaction time of the two-component system (figure 2).

### Phosphomimetic mutant DegS S76D exhibits increased autophosphorylation and phosphotransfer to DegU *in vitro*

**Figure 3:**
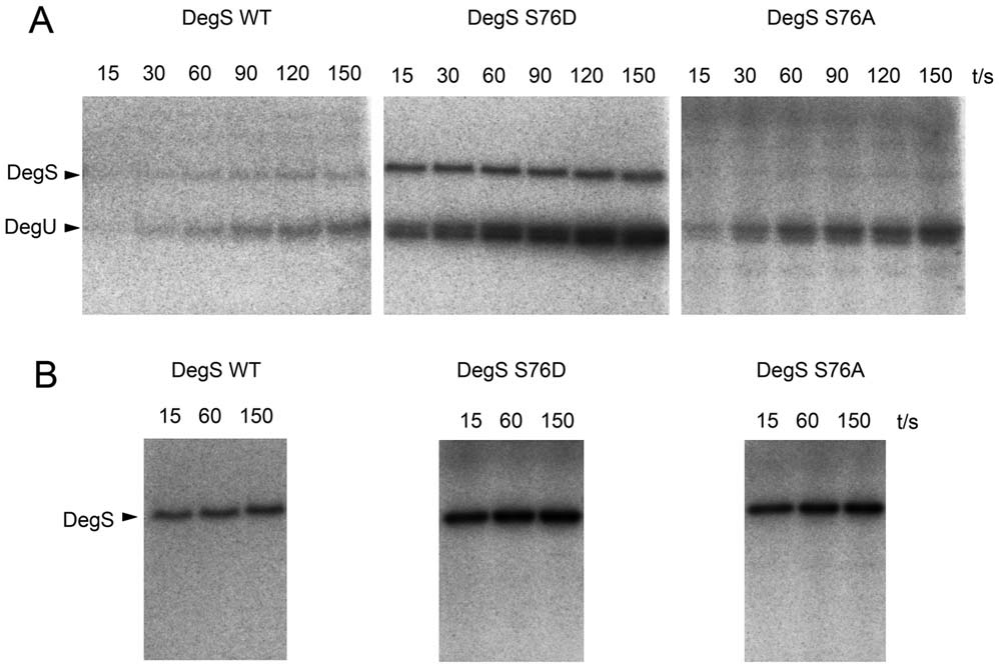
DegS mutations affect autophosphorylation and DegU phosphorylation *in vitro*. Autoradiography of SDS-Polyacrylamide gels with proteins that had been incubated with ^32^P-γ-ATP. Gels were not treated by boiling in acid, so the phospho-histidine and phospho-aspartate signals are preserved. Time periods are indicated for each lane, for DegU phosphorylation (A) and DegS autophosphorylation (B).

Rarely more than several percent of the target protein is phosphorylated during *in vitro* kinase assays, unless a specific kinase-activating signal is present [28]. In order to study the regulatory effects of phosphorylation *in vivo*, phosphomimetic mutants, with the phosphorylatable residue replaced by a larger, negatively charged amino acid are often employed [29]. Since our data indicated that DegS can be phosphorylated by two Hanks-type kinases, for which the specific effectors (or conditions) that trigger their activity towards DegS are unknown, it seemed particularly promising to study the effects of DegS phosphorylation *in vivo* using a phosphomimetic mutant DegS S76D. Before using the mutant protein *in vivo*, we checked whether its behaviour during an *in vitro* phosphorylation assay would corresponded to that of wild type DegS which had been phosphorylated by YbdM (as shown in figure 2). Purified DegS S76D showed an increase in DegU phosphorylation on aspartate (figure 3A) as well as an increase in autophosphorylation on histidine (figure 3B) compared to the wild type. The respective activites of DegS S76A were also above the wild type level, but below that of DegS S76D (figure 3). Interestingly, DegS S76D maintained a considerable level of incorporated phosphate in the presence of DegU, whereas DegS wild type and DegS S76A retained much less phosphate under these conditions. DegS/U exerts a very complex regulation of several physiological processes, some of which are affected by high and others by low levels of DegU phosphorylation. We thus hypothesized that strains where wild type DegS would be replaced by DegS S76D should be unable to activate processes that are normally stimulated by non-phosphorylated DegU, and probably overly stimulate processes that require phosphorylated DegU. We therefore decided to focus on several functions that exemplify these contrasting situations to examine the regulatory role of DegS phosphorylation on serine 76.

### DegS S76D negatively affects competence development

**Figure 4:**
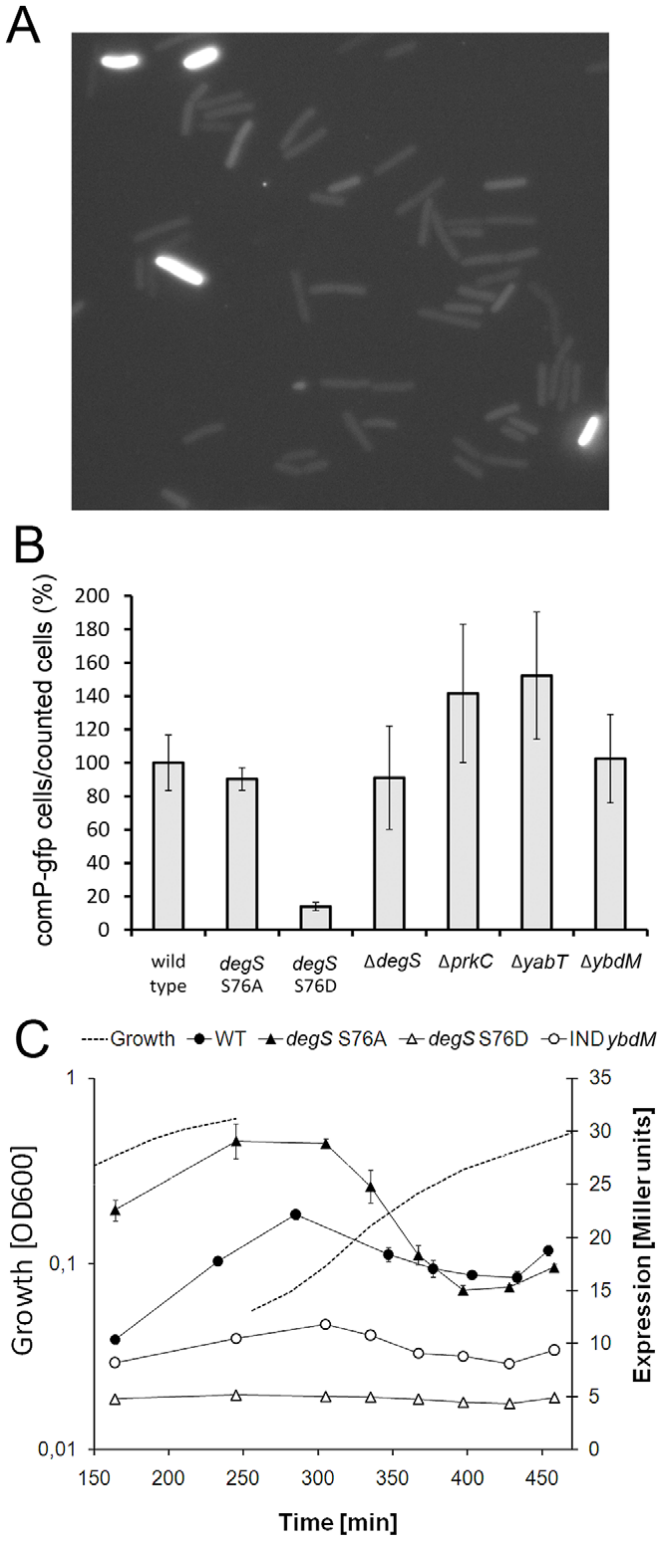
Competence development is inhibited in *degS* S76D strain. (A) Single cell analysis of competence: a representative picture demonstrating the difference in fluorescence intensity observed between competent and non-competent cells. (B) Single cell analysis of competence: numerical data. Competence of wild type, *degS* mutants and Ser/Thr kinase mutants normalised with respect to the wild type cells. The results (with standard deviation bars) are the average of three independent experiments. (C) *comK* promoter activity in wild type, *deg*S S76A, *degS* S76D and a strain overexpressing *ybdM* grown in competence media. Wild type is shown in filled circles, *degS* S76A in filled triangles, *degS* S76D in open triangles and the strain overexpressing *ybdM* in open circles. Culture growth is indicated with the dotted line (broken line indicates the dilution in the new medium). The results (with standard deviation bars) are the average from three technical replicas.

Competence development is a complex process regulated partly by DegS/U via the transcriptional activation of *comK* exerted by unphosphorylated DegU [30]. To test the effect of phosphorylation of DegS serine 76 on this process, strains were constructed in which the chromosomal version of *degS* was replaced by copies encoding either DegS S76D or DegS S76A. Competence of the resulting strains was initially compared to the wild type by using a two-step transformation protocol [31]. The competence was quantified as the number of colonies per µg of plasmid used for transformation, and normalized for the wild type (100+/−12%). The strain *degS* S76A (155+/−7%) was about 50% more competent than the wild type, while the *degS* S76D mutant exhibited an approximately 5-fold reduction in competence (18+/−0%), concurring with our working hypothesis. Next, competence development on single cell level was assayed by introducing GFP under control of the *comK* promoter. In this system we further assayed the effects of inactivating individual serine/threonine kinase-encoding genes *prkC*, *ybdM and yabT* (figure 4). Our hypothesis was confirmed, with an approximate 5-fold competence reduction in *degS* S76D, while in this set up no difference between wild type and the *degS* S76A strain was observed. In this setup, a mutant of the kinase phosphorylating DegS would be expected to behave as the non-phosphorylatable *degS* S76A. All kinase mutants had competence level identical or superior to wild-type, which is in agreement with our hypothesis, but of limited evidential value, since *degS* S76A was itself non-distinguishable from the wild type. Competence in *B. subtilis* is a bistability phenomenon that occurs in a sub-population of cells which become transiently competent during a certain window of time in the transition growth phase [32]. We thus asked the question whether the effect of *degS* S76D on competence could be due to a temporal shift in the window of competence or an overall decrease in DegU-dependent *comK* expression. In order to determine this, *lacZ* encoding β-galactosidase was placed under the control of the *comK* promoter and introduced in the *amyE* locus of wild type and mutant strains. Activity of the *comK* promoter was assayed in the two-step competence media. Expression of *comK* was similar in the wild type and *degS* S76A cells, peaking out as expected in the early transition phase. In the *degS* S76D mutant, expression of *comK* was 5-fold lower, and not induced at all in the transition phase (figure 4C). When we overexpressed *ybdM* from an IPTG-dependent promoter in the wild type strain, *comK* was still induced, but less efficiently than in the wild type. Since *comK* expression is known to be activated by unphosphorylated DegU, the conclusion we reached based on the presented data was that the *degS* S76D mutation indeed leads to an increase in the DegU∼P pool *in vivo*, in accordance to our phosphorylation data obtained *in vitro*.

### DegS S76D affects complex colony formation and swarming

**Figure 5:**
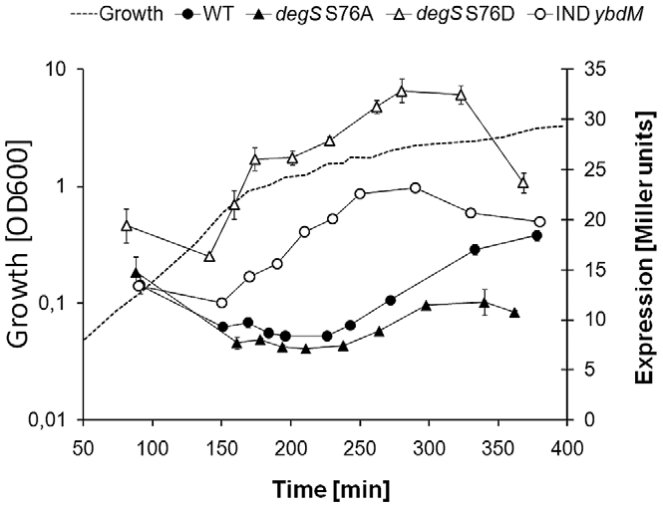
*degS* S76D leads to increased *yvcA* promoter activity. *yvcA* promoter activity in wild type, *deg*S S76A, S76D and the strain overexpressing *ybdM* grown in LB medium. Wild type is shown in filled circles, *degS* S76A strain in filled triangles, *degS* S76D in open triangles and the strain overexpressing *ybdM* in open circles. Culture growth is indicated with the dotted line. The results (with standard deviation bars) are the average from three technical replicas.

After confirming the effect of the phosphomimetic mutation S76D on the pool of DegU∼P using the *comK* promoter, activated by unphosphorylated DegU, we set out to confirm this finding from the opposite angle. We next examined the *yvcA* promoter, recently shown to be activated by intermediate levels of DegU∼P [12]. We used the same experimental setup with promoter-*lacZ* fusions introduced ectopically, and the promoter activities were determined in cells grown in LB medium (figure 5). A significant increase in *yvcA* promoter activity was observed in the *degS* S76D strain and to a lesser extent in the strain overexpressing *ybdM*, compared to the wild type and *degS* S76A strain. A gradual increase in expression from the *yvcA* promoter in the wild type *B. subtilis* compared to the *degS* S76A strain observed in the stationary phase might indicate a gradual increase in the level of DegS serine phosphorylation, which is completely abolished in *degS* S76A. These results further substantiate that the level of DegU∼P is increased in the *degS* S76D mutant. The *yvcA* promoter has been reported to be inhibited at high levels of DegU∼P, indicating that this mutation leads rather to intermediate than excessive amount of DegU∼P. Further substantiating this, protease production, known to be activated by high levels of DegU∼P, was not stimulated by the *degS* S76D mutant (data not shown).

**Figure 6:**
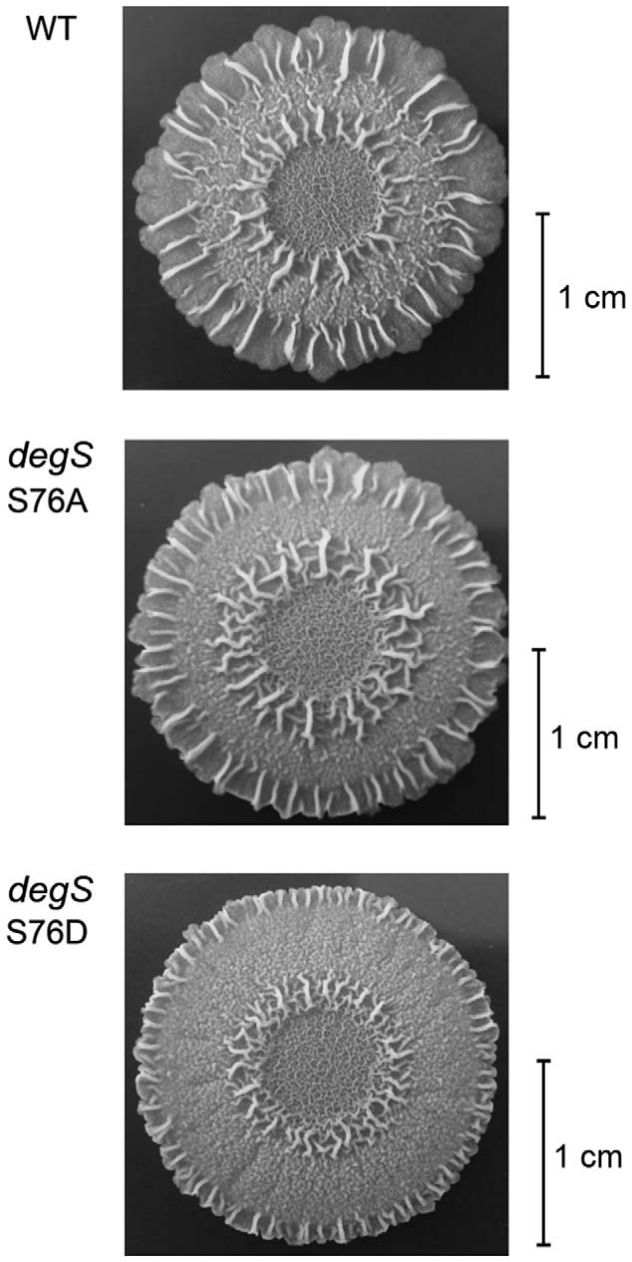
Complex colony formation. Complex colony formation of the wild type *B. subtilis* and mutant strains *degS* S76A and *degS* S76D grown on MSgg medium. Representative colonies are shown, together with a scale bar.

YvcA has been shown to play an important role in complex colony formation [12]. The effects on *yvcA* promoter activity prompted us to investigate the effects of the *degS* S76D mutation on this and the other social behavioural traits pellicle formation and swarming that are regulated by low levels of DegU∼P. The laboratory strain *B. subtilis* 168 does not readily swarm due to defects in surfactin production and a frame shift mutation in *swrA* [33] and we therefore tested these traits in the undomesticated strain NCIB 3610. No effect was observed on pellicle formation (data not shown). Concerning complex colony formation, all strains exhibited some variability on the MSgg medium. However, there was a subtle difference in colony morphology: the wild type and *degS* S76A colonies were capable of producing larger and more complex aerial structures than the strain *deg*S *S*76D (figure 6).

**Figure 7:**
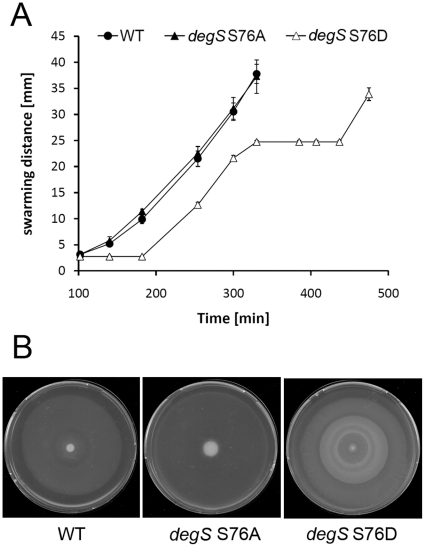
Swarming is affected by *degS* S76D. Swarming of the wild type *B. subtilis* and mutant strains *degS* S76A and *degS* S76D, Swarming speed (A) was followed by measuring the radii of the swarming zones on plates at designated time intervals. Wild type is shown in filled circles, *degS* S76A strain in filled triangles and *degS* S76D in open triangles. Consolidation of swarms (B) was documented one hour after the swarming reached the end of plates (367 min for wild type, 374 min for *degS* S76A, and 502 min for *degS* S76D). One of three independent experiments (all yielding similar results) is shown.

A more apparent phenotype was observed in swarming (figure 7). The wild type and *degS* S76A swarming pattern and speed were similar, they swarmed in a linear manner until reaching the end of the plate in just over 300 min (figure 7A). The *degS* S76D strain swarmed in two phases. After an initial lag (compared to the wild type) of about 60 min, it started swarming at the same speed as the wild type until reaching about one half-distance towards the end of the plate. Then it paused for about 120 min, seemingly consolidating. Finally, it continued swarming and went on to reach the end of the plate with about 180 min delay compared to the wild type. Due to this stop-and-go behaviour, the *degS* S76D final swarm exhibited concentric layers at various stages of consolidation (figure 7B). It is difficult to explain this altered swarming pattern in the strain *degS* S76D. It has previously been reported that expression of the flagella operon is inhibited at high DegU∼P levels [34], but we observed no difference in flagella amount or organisation in the three strains at the time of *degS* S76D consolidation (data not shown). Further, this phenotype does not seem to be related to either surfactin production or the *swrA* mutation since a lab strain cured for these mutations, DS155 [33], exhibited no phenotype on swarming (data not shown). Nevertheless, these data collectively support the idea that *degS* S76D mutation indeed leads to an increased DegU∼P level. If this mutation can mimic the phosphorylated form of DegS *in vivo*, it would indicate that the serine phosphorylation event could have the potential to regulate social behaviours regulated at low DegU∼P levels in *B. subtilis*.

### Concluding remarks

Here we describe, to the best of our knowledge, the first example of a bacterial two component sensory kinase that is regulated via serine phosphorylation of its input domain by a Hanks type Ser/Thr kinase. DegS was phosphorylated by Hanks type serine/threonine kinases YbdM and YabT *in vitro*. YbdM-dependent phosphorylation was specific for serine 76 and it led to increased efficiency of phosphotransfer to DegU. Moreover, *ybdM* overexpression led to a similar (albeit less pronounced) *in vivo* effect on some DegU-controlled promoters as the *degS* S76D phosphomimetic mutation. The *degS* S76D strain exhibited phenotypes corresponding to elevated levels of DegU∼P *in vivo*. The use of point-mutations to mimic phosphorylated and non-phosphorylatable proteins is a common tool employed in protein phosphorylation research but it has a potential pitfall. The ensuing phenotypes may be caused by conformational changes caused by the mutations, which are entirely unrelated to phosphorylation. In case of the DegS S76D mutation, the protein behaves in a similar manner as wild type DegS phosphorylated by YbdM. The fact that the mutant protein exhibits a higher DegU kinase activity is expected since it would correspond to 100% phosphorylation of DegS, which is never achieved by incubating DegS with YbdM *in vitro*. The non-phosphorylatable mutant DegS S76A also exhibits an increased DegU kinase activity *in vitro* but this does not translate to an increased pool of DegU∼P *in vivo*. Whether this could reflect a conformational change of the protein leading to increased kinase activity or maybe increased stability *in vitro* remains elusive. Knocking out the kinase YbdM did not provoke strong phenotypes, possibly due to complementation of its function by remaining Hanks kinases. The overproduction of the regulatory kinase YbdM, which possibly phosphorylates other proteins, could also lead to non-physiologically relevant phenotypes. The fact remains that there is an agreement between the observations with the phosphomimetic DegS S76D and overexpression of YbdM. If DegS S76D indeed can mimick the serine 76-phosphorylated state of DegS, it would suggest that phosphorylation of serine 76 of DegS contributes to an already very complex process of regulating the level of DegU phosphorylation in *B. subtilis*. The molecular mechanism by which serine phosphorylation activates DegS activity remains elusive. The fact that a phosphomimetic mutant DegS S76D behaved in a similar manner would preclude that phospho-transfer from serine to either DegS histidine or DegU aspartate is involved. It would seem plausible that phosphorylation could induce a conformational change thereby stimulating kinase activity, but due to the lack of any structural data for DegS this is merely a speculation. The residue serine 76 of DegS was found to be phosphorylated in the late exponential growth phase [8], but that study was performed on this single growth condition, and hence the temporal window of DegS serine phosphorylation is not known. Despite YbdM arguably being the most specific of the three, the other two Hanks type kinases present in *B. subtilis* can possibly contribute to phosphorylating DegS serine 76 *in vivo* to some extent. These kinases are presently almost completely uncharacterised (except PrkC) and more work will be needed to elucidate specific signals that trigger their expression, activity and substrate specificity. Our results indicate that DegS serine phosphorylation influences DegU phosphorylation *in vivo*, pointing towards a possible role in regulating phenomena such as motility and complex colony formation.

## Materials and Methods

### Bacterial strains and growth conditions

*E. coli* NM522 was used for plasmid propagation in cloning experiments. The chaperone overproducing strain *E. coli* M15 carrying pREP4-GroESL [35] was used for protein synthesis. *B. subtilis* strains used in this study are listed in table 1. *E. coli* and *B. subtilis* cells were grown at 37°C with shaking in LB medium. In addition, *B. subtilis* was grown in competence media for transformation experiments as described [31] and MSgg medium for complex colony experiments [36]. For *E. coli* ampicillin (100 µg/mL), kanamycin (25 µg/mL), tetracycline (8 µg/mL) and for *B. subtilis* erythromycin (5 µg/mL), neomycin (5 µg/mL) and tetracycline (15 µg/mL) were added as appropriate.

**Table 1:** List of *B. subtilis* strains used in this study.

Strain	Description	Reference
168		[36]
168-*degS* NS	*degS* K9stop	This work
168-PcomK	*amyE*::P*comK-lacZ*	This work
168-PcomK-gfp	*amyE*::P*comK-gfp*	This work
168-PcomK-IND ybdM	*ybdM*::pHT315 *amyE*::P*comK-lacZ*	This work
168-PyvcA	*amyE*::P*yvcA-lacZ*	This work
168-PyvcA-IND ybdM	*ybdM*::pHT315 *amyE*::P*yvcA-lacZ*	This work
168-degS S76A	*degS* S76A	This work
168-S76A-PcomK	*degS* S76A *amyE*::P*comK-lacZ*	This work
168-S76A-PcomK-gfp	*degS* S76A *amyE*::P*comK-gfp*	This work
168-S76A-PyvcA	*degS* S76A *amyE*::P*yvcA-lacZ*	This work
168-degS S76D	*degS* S76D	This work
168-S76D-PcomK	*degS* S76D *amyE*::P*comK-lacZ*	This work
168-S76D-PcomK-gfp	*degS* S76D *amyE*::P*comK-gfp*	This work
168.S76D-PyvcA	*degS* S76D *amyE*::P*yvcA-lacZ*	This work
168-ΔdegS PcomK-gfp	Δ*degS*::pMUTIN2 *amyE*::P*comK-gfp*	This work
168-ΔprkC PcomK-gfp	Δ*prkC*::pMUTIN2 *amyE*::P*comK-gfp*	This work
168-ΔybdM PcomK-gfp	Δ*ybdM*::pMUTIN2 *amyE*::P*comK-gfp*	This work
168-ΔyabT PcomK-gfp	Δ*yabT*::pMUTIN2 *amy*E::P*comK-gfp*	This work
3610	*sfp*+ *swrA*+	Bacillus Genetic Stock Center
3610 degS S76A	*sfp*+ *swrA*+ *degS* S76A	This work
3610 degS S76D	*sfp+ swrA+ degS* S76D	This work
DS155	PY79 *sfp+ swrA+*	[33]

### DNA manipulations and strain construction

*B. subtilis* genes *degS*, *degU*, *prkA*, *prkC* (catalytic domain), *yabT* (catalytic domain) and *ybdM* were PCR-amplified using genomic DNA from the strain 168 as template [37]. In order to improve solubility of PrkC and YabT only the cytosolic part containing the active site was used. Point mutations *degS* S76A and S76D were obtained using two partially overlapping mutagenic primers (table 2). The PCR products were inserted in the vector pQE30 (Qiagen) using appropriate restriction sites. For promoter-*lacZ* fusions the promoter regions were PCR-amplified from genomic DNA and inserted between the *Eco*RI and *Bam*HI sites of pDG268-neo [38]. For *comK* promoter-*gfp* fusion, pDG268neo-PcomK was restricted with *Bam*HI and *Pci*I to remove *lacZ*, *gfp* was PCR-amplified using plasmid pFH2191 [39] as template, restricted and ligated with the vector. *B. subtilis* was transformed with the constructs yielding strains with promoter-*lacZ* or -*gfp* fusions inserted in the *amyE* locus using a one-step transformation method [40]. A nonsense mutation of *degS* (K9stop) was constructed using two partially overlapping mutagenic primers. The PCR product and pHT315 [41] were restricted with *Eco*RV and *Pvu*II and the fragments ligated. The resulting vector was devoid of the Gram-positive origin of replication. Upon transformation and integration on the chromosome, the mutation was verified by sequencing. Inactivation of *prkA*, *prkC*, *ybdM* and *yabT* was done using pMUTIN2 [42]. For IPTG-inducible overexpression, *ybdM* was inserted in pHT315 and introduced in *B. subtilis* strains bearing promoter-*lacZ* fusions. The vector pG^+^host8 containing a temperature sensitive origin of replication was used to introduce *degS* S76A and S76D point mutations *in situ*, replacing the chromosomal version of *degS* in *B. subtilis* 168 [43]. *Bam*HI-*Cfr*9I fragments from pQE30-*degS* S76A and S76D containing the mutated gene were inserted into pG^+^host8 between the *Bam*HI and *Cfr*9I sites. *B. subtilis* was transformed with the constructs, plated on tetracycline-containing plates and incubated at the non-replicative temperature 37°C, which selects for integration of the vector on the chromosome by single crossing-over. The transformants were further cultured in liquid LB for loss of plasmid by the second crossing-over event and the chromosomal mutation was verified by sequencing. In our hands the second-crossing over happened with a low frequency indicating that *B. subtilis*, contrary to *Lactococcus lactis*, was not severely affected by a chromosomal copy of pG^+^host8 actively replicating. Point mutations in *B. subtilis* NCIB3610 were introduced by same method except that transformation with pG^+^Host8 was done by PEG treatment of protoplasts [44].

**Table 2:** Primers used in this study. Restriction sites are underlined and changed codons are in bold.

Name	Sequence^1^	Description
DegU fwd	CGCCGCGGATCCATGACTAAAGTAAACATTGTTATT	*Bam*HI
DegU rev	CGCAATGGTACCTTATCTCATTTCTACCCAGCCATTTTT	*Kpn*I
DegS fwd	CGCCGCGGATCCATGAATAAAACAAAGATGGATTCC	*Bam*HI
DegS rev	CGCAATGGTACCTTAAAGAGATAACGGAACCTTAATCAT	*Kpn*I
PrkC fwd	GAAGATCTATGCTAATCGGCAAGCGGATCAGCGGGCG	*Bgl*II
PrkCtrunc rev	AAAACTGCAGTTACAAAACCCACGGCCACTTTTTTCTTTTTGCCG	*Pst*I, amplification of aa 1–333
YabT fwd	GAAGATCTATGATGAACGACGCTTTGACGAGTTTGGC	*Bgl*II
YabTtrunc rev	AAAACTGCAGTTAGATAAGCGTTGTTTCAAATAACCCC	*Pst*I, amplification of aa 1–321
YbdM fwd	CGGGATCCATGGCATTAAAACTTCTAAAAAAACTGC	*Bam*HI
YbdM rev	AAAACTGCAGTTATGTGACCGATTGAATGGCCCG	*Pst*I
YbdM pHT fwd	CGCGGATCCAAAGGAGGAAAACATATGGCATTAAAACTTCTAAAAAAACTGCTATTTGACC	*Bam*HI
YbdM pHT rev	AAAACTGCAGTTATGTGACCGATTGAATGGCCCGGTTTAGATCCTCG	*Pst*I
PrkA fwd	CGGGATCCATGGATATATTAAAGAAAATTGAAAAGTAC	*Bam*HI
PrkA rev	AAAACTGCAGTTATCGGTTCAGCAGGCTGCCG	*Pst*I
RBS-gfp fwd	CGGGATCCAAAGGAGGAAAACATATGTCTAAAGGTGAAGAACTG	*Bam*HI
gfp rev	CCATACATGTTTATTTATACAGCTCATGCATGC	*Pci*I
degS NS1 fwd	CGGATATCATCTCGTGTTCTCCCGCTTC	*Eco*RV, anneals 263 bp upstream of *degS*
degS NS2 rev	GTCCAGCTGTTCATACTGCTGGCGTGACTGC	*Pvu*II, anneals 123 bp inside *degS*
prkC_ MUT_fwd	CCCAAGCTTAAAGATCCTTTTCATCGCTACG	*Hind*III
prkC_MUT_rev	CGCCCGCGGGGTGACCGTGGCGCCTTCTTTGAC	*Sac*II
yabT_MUT_fwd	CCCAAGCTTATGCAATGGAATACATAAAAGGG	*Hind*III
yabT_MUT_rev	CGCCCGCGGTTGAAGCAGCGGGTTTCCTTCG	*Sac*II
ybdM_MUTfwd	CCCAAGCTTGAATTCATCATAGACGGACAGG	*Hind*III
ybdM_MUTrev	CGCCCGCGGCAGCAAGAATAACAGCGTTTCTCC	*Sac*II
S76A fwd	AAACCGTTTA**GCC**GAGGTCAGCCGTAATTTTCA	S76A
S76A rev	GGCTGACCTC**GGC**TAAACGGTTTCTCGCATGGC	S76A
S76D fwd	AAACCGTTTA**GAC**GAGGTCAGCCGTAATTTTCA	S76D
S76D rev	GGCTGACCTC**GTC**TAAACGGTTTCTCGCATGGC	S76D
degS NS1 rev	AATCCAGCAC**TTA**GGAATCCATCTTTGTTTTATTC	K9Stop
degS NS2 fwd	GATGGATTCC**TAA**GTGCTGGATTCTATTTTGATG	K9Stop
PcomK fwd	CGGAATTCTAAAGAATCCCCCCAATGCC	*Eco*RI
PcomK rev	CGCGGATCCGTCTGTTTTCTGACTCATAT	*Bam*HI
PyvcA fwd	CGGAATTCGAACGCCAAGCGGAAATGCC	*Eco*RI
PyvcA rev	CGCGGATCCCCTGTCAGGGCAAGTAATAAG	*Bam*HI

### Protein synthesis and purification

6xHis-tagged proteins were synthesised in the chaperone-overproducing strain *E. coli* M15 carrying pREP4-*groESL*. Cultures were grown shaking at 37°C to OD_600_ 0.5, induced with 1 mM IPTG and grown for an additional 3 hours. Cells were disrupted by sonication and 6xHis-tagged proteins were purified on Ni-NTA columns (Qiagen) according to manufacturer's instruction, desalted with PD-10 columns (GE-Healthcare) and stored in a buffer containing 50 mM Tris-Cl pH 7.5, 100 mM NaCl and 10% glycerol. Protein concentrations were estimated using the Bradford assay (Bio-Rad) with BSA as standard.

### *In vitro* phosphorylation assay

Phosphorylation reactions were performed in a total volume of 30 µl, essentially as described [28], with 180 nM DegS, DegS S76A or DegS S76D and 15 µM DegU. For serine phosphorylation of DegS, reactions contained 10 nM of either PrkA, PrkC (catalytic domain), YabT (catalytic domain) or YbdM (unless otherwise specified in the figure legend). Besides the proteins, the reaction mixture contained 50 µM ^32^P-γ-ATP (20 µCi/mmol), 42.5 mM Tris-Cl (pH 7.5), 5 mM MgCl_2_, 85 mM NaCl and 8.5% glycerol. For PrkC, we varied the pH value of the Tris-Cl buffer to the additional pH values of 7.0, and 8.0. To measure the influence of YbdM on DegS phosphotransfer to DegU, DegS was pre-incubated with YbdM for 3 h, in exactly the same conditions as described above, only with non-labelled ATP. Reactions were started by addition of ATP, incubated at 37°C for 60 min (unless otherwise indicated in figure legends) and stopped by addition of SDS-containing loading buffer. All gels shown in the same figure have the same exposure times. For dephosphorylation assays, after the initial phosphorylation reaction described above, the DegS/YbdM and DegS/YabT reaction mixtures were desalted on a PD-10 column (to remove the ATP), lyophilized and resuspended in the identical reaction mixture as before, but without ATP, and incubated 2 hours at 37°C. The proteins were separated by SDS-PAGE (for separation of PrkC and DegS we used a Tris-tricine gel). For detection of phospho-histidine and phospho-aspartate, the gels were rinsed with water and dried directly, whereas for detection of phospho-serine they were additionally treated in boiling 0.5 M HCl for 10 min. Radioactive signals were visualised using STORM phosphoimager and quantified using the ImageQuant software (GE-healthcare).

### β-galactosidase assay

150 mL LB was inoculated with overnight culture to OD_600_ of 0.02 and grown with shaking at 37°C. IPTG at a final concentration of 0.5 mM was added where appropriate. At time points indicated in the figures, 2 mL samples were taken, spun down (10000 g, 2 min) and cell pellets were stored at −20°C. The pellet was resuspended in 2 mL of Z-buffer (60 mM Na_2_HPO_4_·7H_2_0, 0.04 mM NaH_2_PO4·H_2_0, 10 mM KCl, 1 mM MgSO_4_ and 50 mM β-mercaptoethanol, pH 7.0) and OD_600_ was measured. 1 mL cell suspension was treated with 0.5 mg lysozyme for 5 min at 30°C before adding 8 µL 10% Triton X-100 and incubating for additional 5 min. Reaction was started by addition of 100 µL of 4 mg/mL ONPG and stopped by addition of 1 mL of 0.5 M Na_2_CO_3_. Miller units were calculated as described previously [45].

### Competence assays

To assess the competence of *B. subtilis* strains cells were transformed according to the two-step protocol described by Yasbin *et al.* with the modification that 60 min after dilution in GM2 medium, 0.5 mL culture were transferred to three test tubes containing 1 µg DNA and incubated an additional 90 min before plating. The plasmid pDG268-neo [38] conferring neomycin resistance was used as DNA for competence experiments. Results from three biological replicates are presented as % of competence in wild type and are average and standard deviation of three transformations. For single cell analysis the cultures were incubated for 105 min after dilution in GM2 at which time cultures were concentrated 10 fold and 5 µL were deposited on a polylysine-coated glass slide (Thermo Scientific) and examined using a Xeiss Axioplan microscope equipped with a Kappa ACC 1 condenser, a Zeiss Plan Neofluor 100× objective and a Kappa DX2 HC-FW camera. Images were acquired using Kappa Imagebase Control 2.7.2 software. In the experiments 1600 to 9800 cells per strain were examined. For wild type cells about 5.4% were competent and values are given as % of wild type with standard deviation of two independent experiments.

### Complex colony formation

Cells were grown in LB shaking at 37°C to OD_600_ 0.5 at which point 5 µL culture was spotted on a dried MSgg plate (1.5% agar) and incubated for 96 hours at 28°C. Colonies were measured and photographed using a SONY Cyber-shot DSC-T20 camera with close focus enabled. For each sample, a representative image from 20 examined colonies is presented.

### Swarming

Cells were grown in LB to an OD_600_ of 0.5 at which time LB plates (0.7% agar) dried for 20 min in a fume hood (face up) where inoculated with 5 µL culture and dried an additional 10 minutes. Petri dishes were sealed with parafilm to avoid plates drying out and incubated at 37°C. Swarm radii were measured at the times indicated in the figure. Plates were scanned about 1 hour after the swarm reached the edge of the plate using a standard HP office scanner.
